# Association between overactive bladder and suicidal ideation in US adults: a population-based study

**DOI:** 10.3389/fpsyt.2025.1483684

**Published:** 2025-06-09

**Authors:** Heng Liu, Huqiang Dong, Mingchu Jin, Yu Zhou, Haidong Hao, Yutang Yuan, Hongtao Jia, Junyong Li

**Affiliations:** ^1^ Department of Urology, Renmin Hospital, Hubei University of Medicine, Shiyan, Hubei, China; ^2^ School of Public Health, Ningxia Medical University, Yinchuan, Ningxia, China

**Keywords:** overactive bladder, suicidal ideation, NHANES, OAB, population-based study

## Abstract

**Background:**

Suicidal ideation, a critical public health issue, is notably associated with mental health disorders. Overactive bladder (OAB), a prevalent urological disorder, significantly impacts patients’ quality of life and is associated with mental health problems such as anxiety and depression. This study investigates the association between OAB and suicidal ideation in US adults.

**Methods:**

This population-based cross-sectional study utilized data from six consecutive NHANES datasets (2007-2018). Suicidal ideation was assessed using the ninth item of the Patient Health Questionnaire-9 (PHQ-9), and OAB was identified through a simplified Overactive Bladder Symptom Score (OABSS). Multivariate logistic regression models, Restricted Cubic Splines, and subgroup analyses were used to analyze. The association between OAB and suicidal ideation, adjusting for potential confounders.

**Results:**

Among the 28,085 participants, 3.30% reported suicidal ideation, and 20.39% were identified with OAB. Individuals with suicidal ideation had a significantly higher prevalence of OAB compared to those without suicidal ideation. After adjusting for covariates, each point increase in OABSS was associated with a 15% higher likelihood of suicidal ideation (OR=1.15, 95% CI: 1.10-1.20). Participants with OAB had a 47% increased likelihood of suicidal ideation compared to those without OAB (OR=1.47, 95% CI: 1.26-1.72). Subgroup analysis proved the robustness of the results of this study.

**Conclusion:**

The findings indicate a significant positive association between OAB and suicidal ideation in US adults. These findings underscore the necessity of integrating urological and mental health care to enhance suicide prevention strategies.

## 1 Background

Suicide is death due to purposeful self-injury, and its incidence varies by factors such as gender and age (1). Globally, more than 700,000 people die by suicide each year. In the United States, more than 16 out of every 100,000 people die by suicide annually (2, 3). These numbers may be underestimated due to incomplete registration of deaths and inaccurate statistics (4). Suicide is not only a personal and family tragedy but also places a heavy burden on community and public health resources (5).

Suicidal ideation, the generation of thoughts about ending one’s life or committing suicide, is a precursor to suicide (6). Studies have shown that there is a strong correlation between suicide and suicidal ideation and that individuals with suicidal ideation face a significantly higher risk of suicide (6, 7). Of those with suicidal ideation, 60% will attempt suicide within a year (7). Suicidal ideation among young Americans increased significantly during the COVID-19 epidemic (8). Therefore, it is critical to explore risk factors for suicidal ideation clinically. One of the primary goals of suicide prevention is to identify at-risk populations (9). Suicidal thoughts are substantially common in individuals in general when mental health issues are present (10). Notably, feelings of sadness and anxiety are among the mental health conditions that overactive bladder illness sufferers are more prone to experience (11, 12). However, it is unclear whether overactive bladder disorder is associated with suicidal ideation. Understanding this relationship is important for effective suicide prevention and intervention.

Overactive bladder (OAB) is a urological disorder characterized by urinary urgency, accompanied by urinary frequency, nocturia, and sometimes urge incontinence, but without pathologic changes such as urinary tract infections (13). The prevalence of OAB is closely related to aging, with a rate exceeding 16% among adults in the United States (14). This disease seriously affects the quality of life of patients and adversely impacts mental health and sleep (15, 16). In addition, OAB imposes a financial burden on patients. As the population ages, this disease also puts increasing pressure on public health (17).

Research has indicated a strong correlation between OAB and interpersonal and psychological aspects. Various causes may trigger OAB and lead to psychological problems, which in turn exacerbate the symptoms of OAB, creating a vicious cycle (18, 19). In the general population, mental health problems are significantly associated with suicidal ideation (10). Therefore, people with OAB may be at risk for mental health risks including suicide (20). However, current studies tend to be limited to women or certain specific populations, which may lead to gender and population bias in the findings, thus reducing their reliability. This is due to gender differences in the urinary system and the different pathophysiologic mechanisms of OAB (21, 22). Therefore, there is a need for a larger population-based study to validate the correlation between OAB and suicidal ideation and to fully detail the interactions between the two. This study was a population-based cross-sectional analysis of U.S. adults aged 20 years and older, based on data from the National Health and Nutrition Examination Survey (NHANES). The investigation of the relationship between OAB and suicidal thoughts was the aim of the study.

## 2 Materials and methods

### 2.1 Study population

The National Health and Nutrition Examination Survey (NHANES) is an ongoing project of the National Center for Health Statistics (NCHS) that provides a comprehensive assessment of the health and nutritional status of the U.S. population. NHANES employs a complex stratified sampling methodology to enhance the accuracy and reliability of its representative sample. NHANES collects extensive data on socioeconomic status, demographic characteristics, dietary habits, and health-related information. Trained personnel conduct the data collection to ensure consistency and precision. All NHANES participants sign a written informed consent form, and the survey protocol receives approval from the NCHS Research Ethics Review Board.

**Figure 1:**
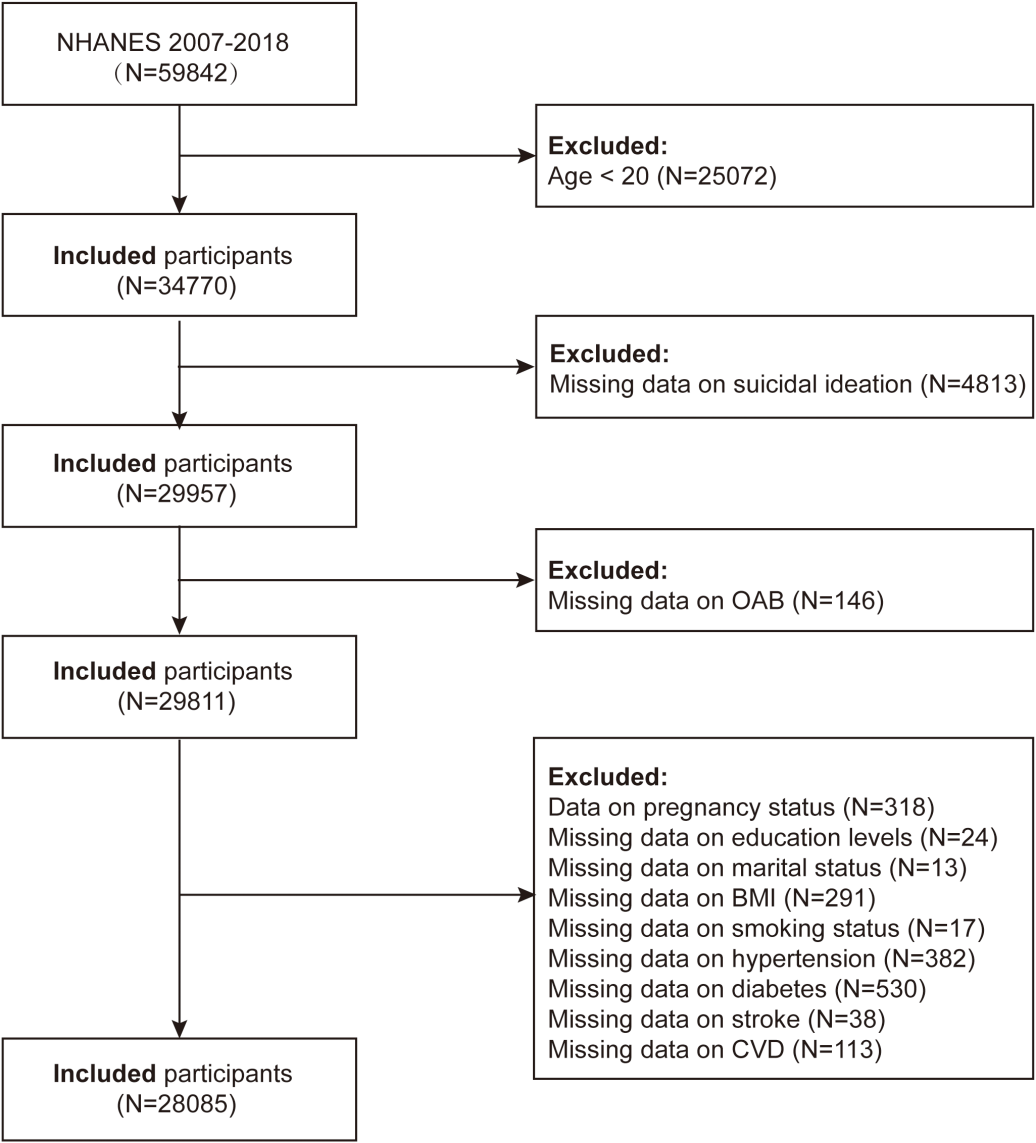
Flowchart of the study population.

In the present study, we downloaded six consecutive datasets (2007-2008, 2009-2010, 2011-2012, 2013-2014, 2015-2016, and 2017-2018) for an accurate evaluation of the connection between OAB and suicidal ideation, consult the NHANES website. We pooled 12 years of data from NHANES. Of the total sample of 59,842 participants, a total of 31,757 were excluded, including data on age <20 years (n=25,072), missing data on OAB (n=146) and suicidal ideation (n=4,813), pregnancy status (n=318), missing data on education (n=24), marital status (n=13), BMI (n=291), smoking (n=17), hypertension (n=382), diabetes (n=530), stroke (n=38), and cardiovascular disease (n=113). The analysis comprised the remaining 28,085 adult US individuals (**Figure 1**).

### 2.2 Definition of suicidal ideation

The ninth item of the Patient Health Questionnaire-9 (PHQ-9) assesses suicidal ideation in the Mental Health Depression Screening section of the survey (23). The PHQ-9 comprises nine items evaluating depressive symptoms experienced over the past two weeks. The ninth item specifically asks, “In the past two weeks, how often have you had thoughts of self-harm or thought it would be better to end your life?” Response options include “Not at all,” “A few days,” “More than half the days,” and “Almost every day.” For this analysis, responses of “Not at all” indicate no suicidal ideation, while all other responses indicate suicidal ideation (24, 25).

### 2.3 Definition of OAB

The International Continence Society (ICS) defines OAB as a syndrome characterized by urinary urgency in the absence of urinary tract infection or other pathology, often accompanied by urinary frequency, nocturia, and urge urinary incontinence (UUI). OAB diagnosis should be considered in patients presenting with UUI or nocturia, and assessed through specific questions regarding the frequency and urgency of urinary leakage. Nocturia burden was evaluated by asking participants how many times they typically got up to urinate at night over the past 30 days. The simplified OABSS was used to measure the severity of OAB, with a score of ≥3 indicating the presence of the disorder, as detailed in **Supplementary Table S1** (26, 27).

### 2.4 Covariates

In this study, covariates included gender, age, race, education level, marital status, household poverty-to-income ratio (PIR), BMI, smoking status, alcohol use, diabetes, hypertension, cardiovascular disease (CVD), and stroke. Individuals in the survey who had smoked at least 100 cigarettes in their lifetime and were smoking at the time of the questionnaire were designated as smokers. Participants who drank at least 12 alcoholic beverages in any year were designated as alcohol drinkers (28, 29). Participants were considered to have diabetes if their physician told them they had diabetes or if their fasting blood glucose was ≥126 mg/dL.

### 2.5 Statistical analysis

All statistical analyses followed the data analysis guidelines provided by NHANES, using appropriate NHANES weights for complex multi-stage cluster sampling designs. The Kolmogorov-Smirnov test was used to check the assumption of normal distribution for each variable. Normally distributed continuous variables were expressed as mean and standard deviation (SD), while non-normally distributed variables were expressed as median and interquartile range (IQR). Categorical variables were expressed as frequency and percentage (%). The study population was categorized into two groups based on the presence or absence of suicidal ideation. The chi-square test, t-test, and Mann–Whitney U test were used to compare the distribution of baseline characteristics between groups.

We used weighted logistic regression to explore the correlation between OAB scores and suicidal ideation. We constructed three logistic regression models. Three statistical models were employed: Model 1 remained unadjusted, Model 2 was adjusted for age, gender, and race, and Model 3 incorporated additional adjustments for education level, marital status, BMI, PIR, smoking, alcohol consumption, diabetes, hypertension, cardiovascular disease, and stroke, along with the covariates in Model 2. We then tested for nonlinear associations between Overactive Bladder Symptom Score (OABSS) and suicidal ideation using restricted cubic spline (RCS) after adjusting for covariates. Finally, subgroup analyses and interaction tests were conducted on the potential confounders listed in the baseline table to explore potential changes in associations between subgroups. Statistical analyses were conducted using R (version 4.3.2), with the significance level set at *P* < 0.05.

## 3 Results

### 3.1 Basic characteristics of the study population

This research comprised 28,085 individuals in total. with 3.30% reporting suicidal ideation. Participants with suicidal ideation were more likely to have OAB (40.28%) and higher OABSS scores compared to those without suicidal ideation (19.71%). The suicidal ideation group was characterized by a higher prevalence of cohabitation with a spouse, high education level, lower PIR level, BMI ≥ 30, hypertension, diabetes, coronary heart disease, stroke, and depression, with significant differences between the groups (P < 0.01) (**Table 1**).

**Table 1:** Characteristics of participants with or without suicidal ideation.

Characteristics	Total (n = 28085)	Non-suicidal ideation (n = 27022)	Suicidal ideation (n = 1023)	*P*-value
Age, years, mean (SD)	49.87 (17.60)	49.87 (17.62)	49.80 (16.90)	0.894
OABSS, median (IQR)	1.00 (0.00,2.00)	1.00 (0.00,2.00)	2.00 (1.00,3.00)	<0.0001
Gender, n (%)				0.1333
Male	14004 (49.39)	13516 (49.48)	488 (46.66)	
Female	14081 (50.61)	13506 (50.52)	575 (53.34)	
Race, n (%)				<0.0001
Mexican American	4204 (8.38)	4025 (8.36)	179 (9.15)	
Other Hispanic	2925 (5.73)	2749 (5.59)	176 (9.85)	
Non-Hispanic White	11793 (67.49)	11374 (67.67)	419 (62.07)	
Non-Hispanic Black	5979 (10.92)	5786 (10.91)	193 (11.43)	
Other Race	3184 (7.47)	3088 (7.47)	96 (7.50)	
Education level, n (%)				<0.0001
Less than high school	6625 (15.06)	6223 (14.67)	402 (26.42)	
High school	6484 (23.16)	6244 (23.12)	240 (24.20)	
More than high school	14976 (61.78)	14555 (62.20)	421 (49.38)	
Marital status, n (%)				<0.0001
Never married	5197 (18.48)	4950 (18.27)	247 (24.71)	
Married/Living with partner	16637 (63.15)	16178 (63.75)	459 (45.49)	
Widowed/divorced/Separated	6251 (18.37)	5894 (17.98)	357 (29.80)	
PIR, n (%)				<0.0001
<1.3	9118 (22.12)	8572 (21.47)	546 (40.91)	
1.3 - 3.5	10289 (34.58)	9939 (34.54)	350 (35.91)	
≥3.5	8678 (43.30)	8511 (43.99)	167 (23.18)	
BMI, kg/m^2^, n (%)				0.0017
<25	7961 (29.30)	7678 (29.35)	283 (27.99)	
25 - 30	9231 (32.87)	8918 (33.04)	313 (28.04)	
≥30	10893 (37.82)	10426 (37.62)	467 (43.97)	
Smoking status, n (%)				<0.0001
Never	15503 (55.22)	15038 (55.60)	465 (44.29)	
Now	5776 (19.77)	5425 (19.31)	351 (33.09)	
Former	6806 (25.01)	6559 (25.09)	247 (22.62)	
Alcohol intake, n (%)				0.6916
No	3305 (10.54)	3196 (10.56)	109 (10.11)	
Yes	24780 (89.46)	23826 (89.44)	954 (89.89)	
Hypertension, n (%)				0.0036
No	15828 (61.50)	15296 (61.69)	532 (55.96)	
Yes	12257 (38.50)	11726 (38.31)	531 (44.04)	
Diabetes, n (%)				<0.0001
No	22954 (86.49)	22147 (86.64)	807 (82.13)	
Yes	5131 (13.51)	4875 (13.36)	256 (17.87)	
Stroke, n (%)				<0.0001
No	27065 (97.26)	26079 (97.38)	986 (93.95)	
Yes	1020 (2.74)	943 (2.62)	77 (6.05)	
CVD, n (%)				0.0018
No	25704 (93.21)	24783 (93.31)	921 (90.19)	
Yes	2381 (6.79)	2239 (6.69)	142 (9.81)	
Depression, n (%)				<0.0001
No	25461 (92.07)	25113 (93.98)	348 (36.09)	
Yes	2538 (7.93)	1831 (6.02)	707 (63.91)	
OAB, n (%)				<0.0001
No	21325 (79.61)	20733 (80.29)	592 (59.72)	
Yes	6760 (20.39)	6289 (19.71)	471 (40.28)	

Weighted.

PIR, the ratio of income to poverty; BMI, body mass index; CVD, cardiovascular disease; OAB, overactive bladder; OABSS, Overactive Bladder Symptom Score; SD, standard deviation; IQR, interquartile range.

### 3.2 Association between overactive bladder and suicidal ideation.

**Figure 2:**
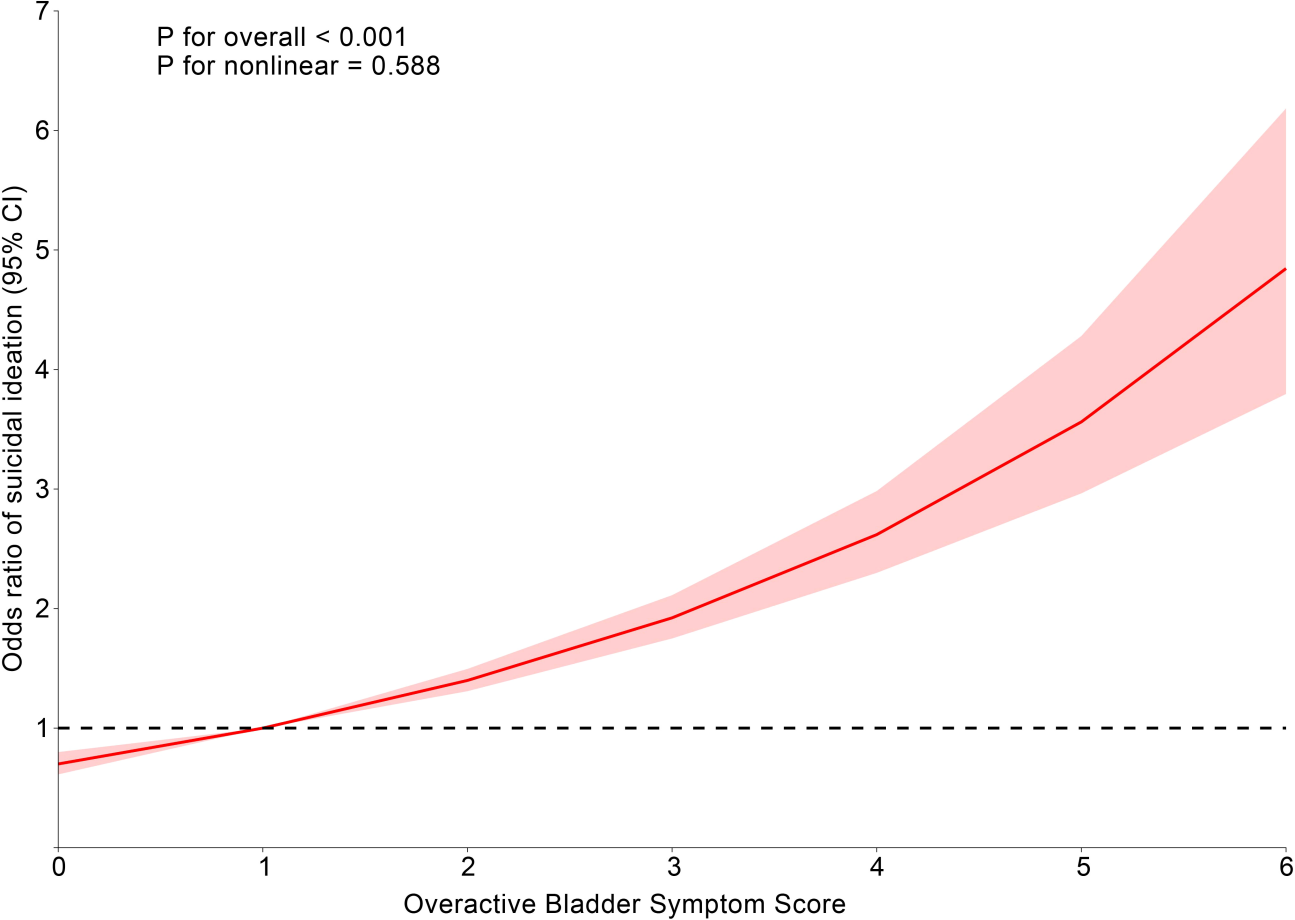
Dose-effect relationship between overactive bladder symptom score and suicidal ideation. Age, gender, race, education level, marital status, PIR, BMI, smoking status, alcohol drinking status, diabetes status, hypertension status, CVD, depression, and stroke were adjusted.

**Table 2** shows the multivariate logistic regression analysis between overactive bladder syndrome and suicidal ideation, which revealed a positive association between OABSS and suicidal ideation. In Model 3, each point increase in OABSS increased the likelihood of participants’ suicidal ideation by 15% (OR = 1.15, 95% CI: 1.10, 1.20). We defined OABSS ≥ 3 points as OAB. Participants with OAB had a 47% increased likelihood of suicidal ideation compared to non-OAB participants (OR = 1.47, 95% CI: 1.26, 1.27) (**Table 2**). Additionally, restricted cubic spline analysis showed no nonlinear association between the Overactive Bladder Symptom Score and suicidal ideation (*P* for nonlinear = 0.588) (**Figure 2**).

**Table 2:** Multiple logistic regression between overactive bladder syndrome and suicidal ideation.

Characteristic	Model 1		Model 2		Model 3	
	OR (95% CI)	*P*-value	OR (95% CI)	*P*-value	OR (95% CI)	*P*-value
OABSS	1.37 (1.32, 1.42)	<0.0001	1.48 (1.42, 1.54)	<0.0001	1.15 (1.10, 1.20)	<0.0001
OAB						
No	1(ref)		1(ref)		1(ref)	
Yes	2.62 (2.32, 2.97)	<0.0001	3.05 (2.66, 3.50)	<0.0001	1.47 (1.26, 1.72)	<0.0001

Model 1: No covariates were adjusted.

Model 2: Age, gender, and race were adjusted.

Model 3: Age, gender, race, education level, marital status, PIR, BMI, smoking status, alcohol drinking status, diabetes status, hypertension status, CVD, depression, and stroke were adjusted.

### 3.3 Subgroup analysis

**Figure 3:**
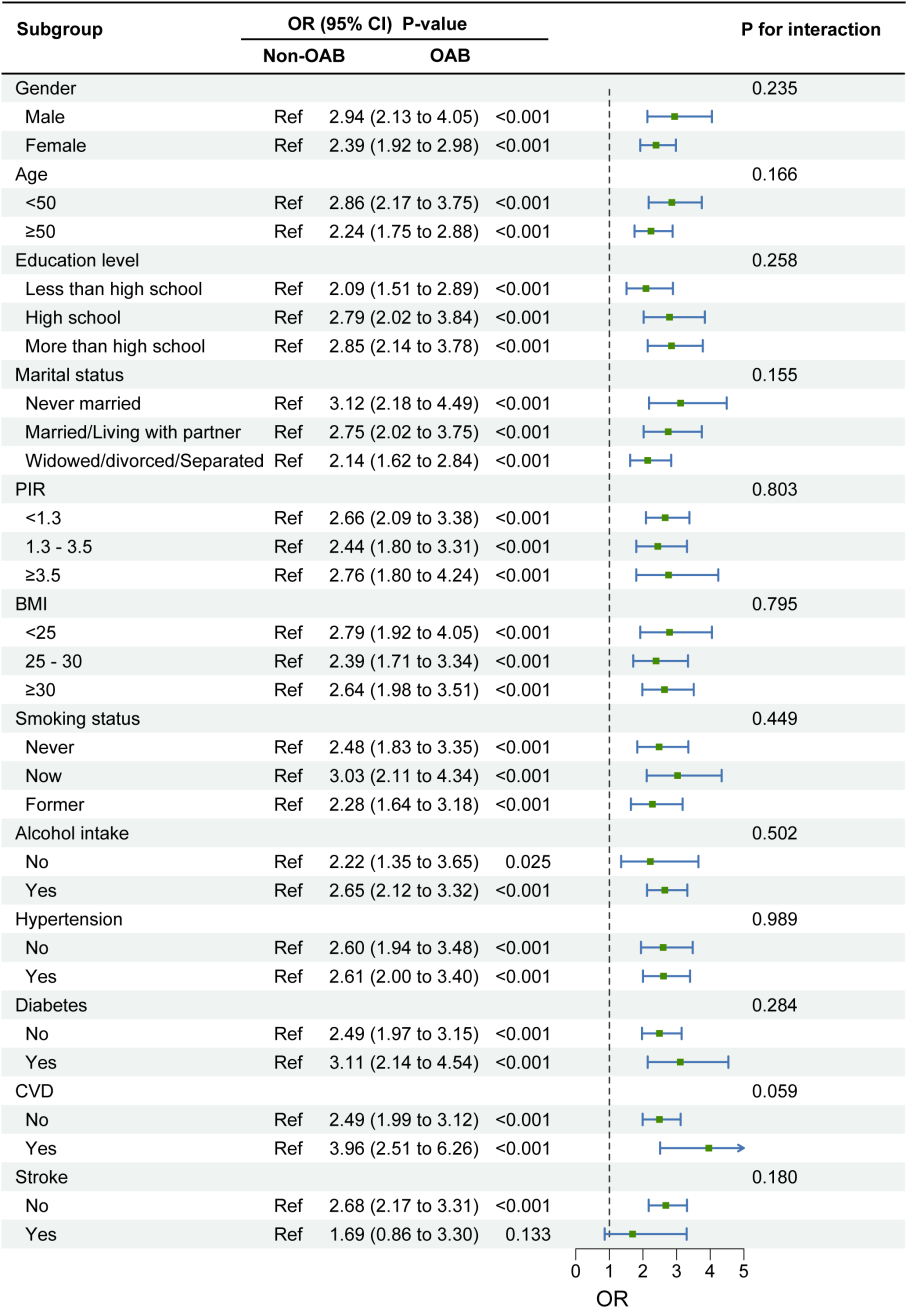
Subgroup analysis of the association between overactive bladder syndrome and suicidal ideation. Note 1: The above model adjusted for gender, age, race, education level, marital status, PIR, BMI, smoking status, alcohol drinking status, diabetes status, hypertension status, CVD, depression, and stroke. Note 2: In each case, the model is not adjusted for the stratification variable.

As shown in **Figure 3**, there was a positive correlation between OAB and suicidal ideation in all subgroups, and this positive correlation was not significantly different across subgroups (p for interaction > 0.05). In the gender subgroup, there was a trend of increasing suicidal ideation in both males [OR = 2.94 (95% CI: 2.13, 4.05), *p* < 0.001] and females [OR = 2.39 (95% CI: 1.92, 2.98), *p* < 0.001]. There was a trend of increasing suicidal ideation in both the 20–50 age group [OR = 2.86 (95% CI: 2.17, 3.75), *p* < 0.001] and the >50 age group [OR = 2.24 (95% CI: 1.75, 2.88), *p* < 0.001] (**Figure 3**).

## 4 Discussion

Using NHANES data, the present research investigated the link between suicidal thoughts and overactive bladder (OAB), offering fresh perspectives on the connection between urologic conditions and psychological conditions. The findings indicated that in contrast to people without OAB, those with OAB had a greater incidence of suicidal thoughts, demonstrating the potential impact of OAB on mental health. The robustness of these findings highlights several key points. First, psychiatrists should focus on the impact of urinary symptoms on suicide risk to improve treatment adherence and effectiveness. A comprehensive approach that integrates both urological symptom management and psychological evaluation is essential when treating patients with OAB. Addressing mental health issues such as anxiety, depression, and suicidal ideation may improve treatment adherence, quality of life, and overall prognosis. Therefore, while routine psychological evaluation may not be necessary for all patients with OAB, it is advisable to consider mental health screening in individuals presenting with severe urinary symptoms, low treatment compliance, or signs of psychological distress.

Previous research has concentrated on the relationship between OAB and depression, frequently using samples from hospitals or particular groups of women (30, 31). These studies compared psychological conditions between two groups by dividing the population into case and control groups but were slightly less persuasive due to the limitations of the research methodology. Nonetheless, studies agree that OAB has a strong correlation with the development of depression (32). First, according to a study of a community-based population assessed using a depression scale, a significant correlation was found between depressive symptoms and OAB, suggesting that an elevated risk factor for incontinence in the urine was depressed symptoms (33). Second, Debra et al. demonstrated in their study that there is a significant association between OAB and the development of depressive symptoms (34). Third, one large population-based study found that the risk of developing depression increased as the severity of OAB increased (35). However, this study was conducted via a web-based survey and disregarded the significance of in-person interviews with doctors, and did not control for covariates. In contrast, the present study analyzed data for U.S. adults, effectively controlled for covariates, and improved the credibility and feasibility of the findings.

The relationship between overactive bladder (OAB) and mental health is likely bidirectional. On one hand, psychological distress may contribute to altered bladder function, and on the other, the burden of OAB symptoms may lead to mental health deterioration. Preclinical studies have shown that psychological stress may affect bladder contractility through pathways such as the Rho-kinase (ROCK) signaling cascade, leading to increased voiding frequency and detrusor overactivity. Stress-induced alterations in gut microbiota have also been hypothesized to influence bladder function. Additionally, anxiety and depression may contribute to heightened somatic sensitivity and lower perception thresholds, possibly exacerbating OAB symptoms (20, 36). Conversely, the social and emotional impact of OAB—such as embarrassment, sleep disturbances, reduced work productivity, and social withdrawal—may predispose individuals to depressive symptoms and suicidal ideation (19, 37, 38).

However, it is important to clarify that we do not advocate for psychology as the primary treatment for OAB or as a replacement for conventional urological interventions. OAB is primarily a bladder dysfunction, and treatment should remain focused on behavioral therapies, pharmacological interventions, and, when necessary, surgical options. However, mental health issues, particularly anxiety and depression, may exacerbate OAB symptoms and negatively impact treatment adherence and quality of life. Therefore, while the primary focus of OAB treatment should remain within the urological domain, we suggest that for individuals with significant emotional distress, poor treatment compliance, or reduced quality of life, considering integrated psychological support may improve overall health outcomes and treatment effectiveness.

## 5 Strengths and limitations

This study has several noteworthy strengths. First, it evaluated a large sample of 28,085 participants, each of whom provided comprehensive clinically informative data. The data collection underwent a rigorous quality assurance process to ensure its reliability. Second, the study conducted subgroup analyses based on common co-morbidities. Subgroup and sensitivity analyses were also carried out to strengthen the reliability of the findings. This thorough examination emphasizes how crucial it is for psychiatrists to concentrate on patients’ symptoms connected to the urinary system.

Although NHANES provides a large, nationally representative sample with standardized data collection procedures, it has several limitations that must be acknowledged. First, it is a cross-sectional survey, which limits the ability to infer temporal or causal relationships between OAB and suicidal ideation. Second, the assessment of both OAB and suicidal ideation is based on self-report questionnaires rather than clinical diagnosis, which may lead to misclassification or underreporting. Third, the data lack granularity on the duration, severity, or context of symptoms, which could influence interpretation. Fourth, although we defined OAB as an OABSS ≥3 in accordance with prior NHANES-based literature, this threshold may inadvertently include individuals whose urinary symptoms stem from other causes, such as nocturnal polyuria, sleep apnea, metabolic or behavioral factors, or anatomical abnormalities. Fifth, relying solely on an OABSS ≥3 cutoff to define OAB may lead to misclassification. Future studies should consider incorporating more nuanced symptom-based subtyping and physiological markers to improve the accuracy and clinical utility of OAB phenotyping. Sixth, while NHANES utilizes a multi-stage, stratified probability sampling method to ensure national representativeness, certain subpopulations (e.g., individuals with severe OAB or hospitalized patients) may not be adequately represented. This could limit the generalizability of findings to these specific groups. Additionally, the reliance on self-reported OAB symptoms may not fully capture the clinical complexity or underlying physiology of OAB, thus affecting the precision of the data in elucidating OAB’s impact. These limitations warrant caution when extrapolating our findings to clinical settings or guiding intervention strategies.

## 6 Conclusion

This study reveals a positive association between OAB and suicidal ideation in U.S. adults, emphasizing the need for an integrated clinical approach to address both urinary and mental health symptoms to enhance suicide prevention efforts.

## Data Availability

The original contributions presented in the study are included in the article/**Supplementary Material**. Further inquiries can be directed to the corresponding authors.

## References

[1] Qing G Deng W Zhou Y Zheng L Wang Y Wei B. The association between non-high-density lipoprotein cholesterol to high-density lipoprotein cholesterol ratio (NHHR) and suicidal ideation in adults: a population-based study in the United States. Lipids Health Dis. (2024) 23:17. doi: 10.1186/s12944-024-02012-4 38218917 PMC10788025

[2] Huang D Zhong S Yan H Lai S Lam M Jia Y. Association between serum zinc levels and suicidal ideation in US adults: A population-based cross-sectional study. J Affect Disord. (2023) 329:359–68. doi: 10.1016/j.jad.2023.02.039 36801424

[3] Liang JH Ge WX Jin ZG Wang C Liu ML Pu YQ. Zhang YS et al: Sexual orientation disparities in the prevalence of suicidal ideation among U.S adults aged 20 to 59 years: Results from NHANES 2005-2016. Psychiatry Res. (2024) 331:115639. doi: 10.1016/j.psychres.2023.115639 38039649

[4] Wang P Wang Y Jia X. Association between fecal incontinence and suicidal ideation in adult Americans: Evidence from NHANES 2005-2010. J psychosomatic Res. (2023) 170:111377. doi: 10.1016/j.jpsychores.2023.111377 37229822

[5] Daurio AM Ennis CR Duffy ME Taylor J. A comparative study of suicidal and nonsuicidal self-injury characteristics in heterosexual versus sexual minority females. Psychiatry Res. (2022) 309:114421. doi: 10.1016/j.psychres.2022.114421 35121340

[6] Hubers AAM Moaddine S Peersmann SHM Stijnen T van Duijn E van der Mast RC. Suicidal ideation and subsequent completed suicide in both psychiatric and non-psychiatric populations: a meta-analysis. Epidemiol Psychiatr Sci. (2018) 27:186–98. doi: 10.1017/s2045796016001049 PMC6998965 27989254

[7] Nock MK Borges G Bromet EJ Alonso J Angermeyer M Beautrais A. Gluzman S et al: Cross-national prevalence and risk factors for suicidal ideation, plans and attempts. Br J psychiatry: J Ment Sci. (2008) 192:98–105. doi: 10.1192/bjp.bp.107.040113 PMC2259024 18245022

[8] Bersia M Koumantakis E Berchialla P Charrier L Ricotti A Grimaldi P. Suicide spectrum among young people during the COVID-19 pandemic: A systematic review and meta-analysis. EClinicalMedicine. (2022) 54:101705. doi: 10.1016/j.eclinm.2022.101705 36338787 PMC9621691

[9] Hawton K van Heeringen K. Suicide. Lancet (London England). (2009) 373:1372–81. doi: 10.1016/s0140-6736(09)60372-x 19376453

[10] Lawrence HR Balkind EG Ji JL Burke TA Liu RT. Mental imagery of suicide and non-suicidal self-injury: A meta-analysis and systematic review. Clin Psychol Rev. (2023) 103:102302. doi: 10.1016/j.cpr.2023.102302 37329877 PMC10330912

[11] Zhang Y Wu X Liu G Feng X Jiang H Zhang X. Association between overactive bladder and depression in American adults: A cross-sectional study from NHANES 2005-2018. J Affect Disord. (2024) 356:545–53. doi: 10.1016/j.jad.2024.04.030 38642902

[12] Vrijens D Drossaerts J van Koeveringe G Van Kerrebroeck P van Os J Leue C. Affective symptoms and the overactive bladder - a systematic review. J psychosomatic Res. (2015) 78:95–108. doi: 10.1016/j.jpsychores.2014.11.019 25499886

[13] Abrams P Cardozo L Fall M Griffiths D Rosier P Ulmsten U. The standardisation of terminology in lower urinary tract function: report from the standardisation sub-committee of the International Continence Society. Urology. (2003) 61:37–49. doi: 10.1016/s0090-4295(02)02243-4 12559262

[14] Wei B Zhao Y Lin P Qiu W Wang S Gu C. The association between overactive bladder and systemic immunity-inflammation index: a cross-sectional study of NHANES 2005 to 2018. Sci Rep. (2024) 14:12579. doi: 10.1038/s41598-024-63448-3 38822015 PMC11143340

[15] Stewart WF Van Rooyen JB Cundiff GW Abrams P Herzog AR Corey R. Prevalence and burden of overactive bladder in the United States. World J Urol. (2003) 20:327–36. doi: 10.1007/s00345-002-0301-4 12811491

[16] Kinsey D Pretorius S Glover L Alexander T. The psychological impact of overactive bladder: A systematic review. J Health Psychol. (2016) 21:69–81. doi: 10.1177/1359105314522084 24591118

[17] Reynolds WS Fowke J Dmochowski R. The burden of overactive bladder on US public health. Curr bladder dysfunction Rep. (2016) 11:8–13. doi: 10.1007/s11884-016-0344-9 PMC4821440 27057265

[18] Jin Z Zhang Q Yu Y Zhang R Ding G Li T. Progress in overactive bladder: novel avenues from psychology to clinical opinions. PeerJ. (2023) 11:e16112. doi: 10.7717/peerj.16112 37927797 PMC10625349

[19] Sexton CC Coyne KS Thompson C Bavendam T Chen CI Markland A. Prevalence and effect on health-related quality of life of overactive bladder in older americans: results from the epidemiology of lower urinary tract symptoms study. J Am Geriatrics Soc. (2011) 59:1465–70. doi: 10.1111/j.1532-5415.2011.03492.x 21718275

[20] Zhang X Ma L Li J Zhang W Xie Y Wang Y. Mental health and lower urinary tract symptoms: Results from the NHANES and Mendelian randomization study. J psychosomatic Res. (2024) 178:111599. doi: 10.1016/j.jpsychores.2024.111599 38309129

[21] Bradley CS Nygaard IE Hillis SL Torner JC Sadler AG. Longitudinal associations between mental health conditions and overactive bladder in women veterans. Am J obstetrics gynecology. (2017) 217:430. doi: 10.1016/j.ajog.2017.06.016 28645572

[22] Buford K Jivanji D Polland A. Microhematuria in women presenting for overactive bladder. Curr Urol Rep. (2023) 24:25–32. doi: 10.1007/s11934-022-01128-3 36445613

[23] Kroenke K Spitzer RL Williams JB. The PHQ-9: validity of a brief depression severity measure. J Gen Internal Med. (2001) 16:606–13. doi: 10.1046/j.1525-1497.2001.016009606.x PMC1495268 11556941

[24] Han KM Ko YH Shin C Lee JH Choi J Kwon DY. Tinnitus, depression, and suicidal ideation in adults: A nationally representative general population sample. J Psychiatr Res. (2018) 98:124–32. doi: 10.1016/j.jpsychires.2018.01.003 29406247

[25] Tan MY Wu S Zhu SX Jiang LH. Association between exposure to organophosphorus pesticide and suicidal ideation among U.S. adults: A population-based study. Ecotoxicology Environ Saf. (2024) 281:116572. doi: 10.1016/j.ecoenv.2024.116572 38896903

[26] Zhu S Wang Z Tao Z Wang S Wang Z. Relationship between marijuana use and overactive bladder (OAB): A cross-sectional research of NHANES 2005 to 2018. Am J Med. (2023) 136:72–8. doi: 10.1016/j.amjmed.2022.08.031 36150516

[27] Tang F Zhang J Huang R Zhou H Yan T Tang Z. The association between wet overactive bladder and consumption of tea, coffee, and caffeine: Results from 2005–2018 National Health and Nutrition Examination Survey. Clin Nutr (Edinburgh Scotland). (2024) 43:1261–9. doi: 10.1016/j.clnu.2024.03.027 38653009

[28] Lv J Xu T Lou S Zhan Z Cheng Z Fu F. Association between serum β-carotene and suicidal ideation in adults: a cross-sectional study. Front Nutr. (2024) 11:1500107. doi: 10.3389/fnut.2024.1500107 39749353 PMC11693589

[29] Huang H Fu J Lu K Fu Y Zhuge P Yao Y. Association between dietary fiber intake and suicidal ideation: a cross-sectional survey. Front Nutr. (2024) 11:1465736. doi: 10.3389/fnut.2024.1465736 39539370 PMC11557476

[30] Bradley CS Nygaard IE Torner JC Hillis SL Johnson S Sadler AG. Overactive bladder and mental health symptoms in recently deployed female veterans. J Urol. (2014) 191:1327–32. doi: 10.1016/j.juro.2013.11.100 24316095

[31] Lai HH Rawal A Shen B Vetter J. The relationship between anxiety and overactive bladder or urinary incontinence symptoms in the clinical population. Urology. (2016) 98:50–7. doi: 10.1016/j.urology.2016.07.013 PMC5116264 27450939

[32] Lai H Gardner V Vetter J Andriole GL. Correlation between psychological stress levels and the severity of overactive bladder symptoms. BMC Urol. (2015) 15:14. doi: 10.1186/s12894-015-0009-6 25887525 PMC4357155

[33] Ikeda Y Nakagawa H Ohmori-Matsuda K Hozawa A Masamune Y Nishino Y. Risk factors for overactive bladder in the elderly population: a community-based study with face-to-face interview. Int J urology: Off J Japanese Urological Assoc. (2011) 18:212–8. doi: 10.1111/j.1442-2042.2010.02696.x 21198945

[34] Irwin DE Milsom I Kopp Z Abrams P Artibani W Herschorn S. Prevalence, severity, and symptom bother of lower urinary tract symptoms among men in the EPIC study: impact of overactive bladder. Eur Urol. (2009) 56:14–20. doi: 10.1016/j.eururo.2009.02.026 19278775

[35] Lee KS Yoo TK Liao L Wang J Chuang YC Liu SP. Association of lower urinary tract symptoms and OAB severity with quality of life and mental health in China, Taiwan and South Korea: results from a cross-sectional, population-based study. BMC Urol. (2017) 17:108. doi: 10.1186/s12894-017-0294-3 29162085 PMC5698954

[36] Mills KA West EG Sellers DJ Chess-Williams R McDermott C. Psychological stress induced bladder overactivity in female mice is associated with enhanced afferent nerve activity. Sci Rep. (2021) 11:17508. doi: 10.1038/s41598-021-97053-5 34471159 PMC8410840

[37] Coyne KS Sexton CC Irwin DE Kopp ZS Kelleher CJ Milsom I. The impact of overactive bladder, incontinence and other lower urinary tract symptoms on quality of life, work productivity, sexuality and emotional well-being in men and women: results from the EPIC study. BJU Int. (2008) 101:1388–95. doi: 10.1111/j.1464-410X.2008.07601.x 18454794

[38] Gomes CM Averbeck MA Koyama M Soler R. Impact of OAB symptoms on work, quality of life and treatment-seeking behavior in Brazil. Curr Med Res Opin. (2020) 36:1403–15. doi: 10.1080/03007995.2020.1760806 32329367

