# Supplementary material for: Association between overactive bladder and suicidal ideation in US adults: a population-based study
Source: Front Psychiatry. 2025 Jun 9;16:1483684. doi: 10.3389/fpsyt.2025.1483684 (PMC12183280; doi:10.3389/fpsyt.2025.1483684)
Supplement: Supplementary file 1 [file Table1.docx]

**Supplementary Table 1** **Criteria for Conversion of Symptom Frequencies recorded in NHANES and OABSS Scores**

| **According to NHANES Score** | **According to OABSS Score** |
| --- | --- |
| Urge urinary incontinence frequency | Urge urinary incontinence score |
| Never | 0 |
| Less than once a month | 1 |
| A few times a month | 1 |
| A few times a week | 2 |
| Every day or night | 3 |
| Nocturia frequency | Nocturia score |
| 0 | 0 |
| 1 | 1 |
| 2 | 2 |
| 3 | 3 |
| 4 | 3 |
| 5 or more | 3 |
| When total score ≥3, the diagnosis is OAB | |

NHANES = National Health and Nutrition Examination Survey; OABSS = Overactive Bladder Symptom Score
